# Health Literacy in Health Professionals Two Years into the COVID-19 Pandemic: Results From a Scoping Review

**DOI:** 10.2196/39023

**Published:** 2022-10-17

**Authors:** Eva-Maria Grepmeier, Maja Pawellek, Janina Curbach, Julia von Sommoggy, Karl Philipp Drewitz, Claudia Hasenpusch, Eva Maria Bitzer, Christian Apfelbacher, Uwe Matterne

**Affiliations:** 1 Institute of Social Medicine and Health Systems Research Otto von Guericke University Magdeburg Magdeburg Germany; 2 University Children’s Hospital Regensburg University of Regensburg Regensburg Germany; 3 Department of Business Studies Ostbayerische Technische Hochschule Regensburg Regensburg Germany; 4 Medical Sociology Institute for Epidemiology and Preventive Medicine University of Regensburg Regensburg Germany; 5 Department of Public Health and Health Education Freiburg University of Education Freiburg Germany

**Keywords:** SARS-CoV-2, COVID-19, health competence, COVID-19–related health literacy, health care worker

## Abstract

**Background:**

Health literacy (HL) is an important public health goal but also crucial in individuals providing medical care. During the pandemic, COVID-19–related HL of health professionals (HPs) has gained momentum; it helps to minimize the risk of self-infection, on the one hand, and to protect patients and relatives from infection, on the other. However, comprehensive information about the levels of individual pandemic-related HL in HPs is scarce.

**Objective:**

In this paper, we aimed at describing the extent of existing research on HL (concept) conducted in HPs (population) in the COVID-19 pandemic (context). The review intends to map the literature on HL in HPs, thereby highlighting research gaps.

**Methods:**

This scoping review was conducted using the methodology of Khalil et al (2016). This involved an electronic search of PubMed (MEDLINE) and PsycInfo and a hand search. The included studies were iteratively examined to find items representing the four HL dimensions of *access*, *understand*, *critically appraise*, and *apply* COVID-19–related health information.

**Results:**

The search yielded a total of 3875 references. Only 7 (1.4%) of the 489 included studies explicitly stated to have addressed HL; 2 (0.4%) studies attempted to develop an instrument measuring COVID-19–related HL in HPs; 6 (1.2%) studies included an HL measure in an observational survey design. Of the remainder, the vast majority used a cross-sectional design. The dimensions *access* and *understand* were frequently examined, but few studies looked at the dimensions *critical appraisal* or *apply*. Very few studies reported an intervention aiming to improve a COVID-19–related HL outcome.

**Conclusions:**

High levels of COVID-19–related HL among HPs are necessary to ensure not only safe practice with necessary protection of HPs, their patients, and relatives, but also successful care delivery and subsequently improved health outcomes in the long term. To advance our understanding of how high COVID-19–related HL manifests itself in HPs, how it relates to health outcomes, and how it can be improved, more research is necessary.

**Trial Registration:**

Open Science Framework dbfa5; https://osf.io/dbfa5/

## Introduction

### Background

Since late 2019, the world has been challenged by a new coronavirus, SARS-CoV-2. Besides its health, economic, social, and psychological impact [1], the pandemic has posed unprecedented challenges, particularly for health professionals (HPs) [2]. Many HPs are in a particularly exposed position during a pandemic [2]. Being in direct contact with COVID-19–infected patients in intensive care units or COVID-19 wards, or as general or specialist practitioners continuing to provide a safe service to patients, requires adaptation to new daily routines and workloads. HPs need to provide care for infected patients, continue to provide the necessary care for noninfected patients, and make sure not to infect patients nor themselves, their family, or significant others. In addition, HPs have a major societal responsibility to stop or mitigate the spread of the pandemic. They are not only required to provide health care services to patients [3] in their actions, but they must also consider their own, their patients’, and their family’s health [4]. HPs represent a population of individuals who are at an increased risk of infection due to the setting in which they work, and simultaneously they could pose a high risk to others due to high frequency of contacts. Therefore, their pandemic-related behavior is crucial to protect themselves and others from infection.

While guidance on how to organize these routines is provided by governmental policies and professional organizations, HPs may still face difficulty in meeting the many new demands placed on them during a pandemic [4]. HPs may also feel at the core of a dilemma. While encountering the ethical responsibility and moral obligation to spend time in places and situations where infection is more likely, they may also feel the concurrent need to protect themselves from infection [5,6]. The majority is willing to go to work [7,8], but the decision appears to be influenced by the preparedness of the organization [9] and other factors [8]. Sufficient availability of evidence-based protective measures, including personal protective equipment (PPE), is at the core of a health care facility’s preparedness [10].

In all these and many other scenarios, the concept of health literacy (HL) can be considered as a key aspect for HPs’ ability to adequately deal with a pandemic’s ubiquitous demands and challenges. HL “represents the cognitive and social skills which determine the motivation and ability of individuals to gain access to, understand and use information in ways which promote and maintain good health” [11]. It can also be understood to entail “the motivation, knowledge and competencies to access, understand, appraise and apply health information in order to make judgements and take decisions in everyday life concerning health care, disease prevention and health promotion to maintain or improve quality of life throughout the course of life” [12]. COVID-19–related HL can be understood as the level or extent of knowledge, motivation, and abilities of individuals to find, understand, and appraise pandemic-related health information and apply the results when making COVID-19–related health decisions. This includes, for example, knowledge about the application of measures to prevent COVID-19 infections, including vaccination-related aspects, detecting infections at an early stage (eg, through regular testing), and seeking medical assistance in case of a positive test or symptoms. Especially in a pandemic situation, which the world has been facing since 2019, one is dealing with a very rapidly changing evidence landscape. This makes it more important to find out what skills people working in the health sector have in terms of information access and understanding, information appraisal, and application.

Often, it is the HL of the general population that matters, because it has been established that HL is not only associated with a range of health outcomes [13,14] but also with social determinants of health. Large parts of the population report difficulties in accessing, understanding, appraising, and applying general health information [15-17], which is an important point, especially considering that compliance to infection prevention measures by each individual is critical in mitigating pandemics. However, comprehensive information about the levels of individual pandemic–related HL in HPs is scarce.

### Objectives

It was the aim of this scoping review to describe the extent of research on HL (concept), conducted in HPs (population) in the COVID-19 pandemic (context). The review intends to map the literature on HL in HPs, thereby highlighting research gaps.

## Methods

### Overview

**Figure 1:**
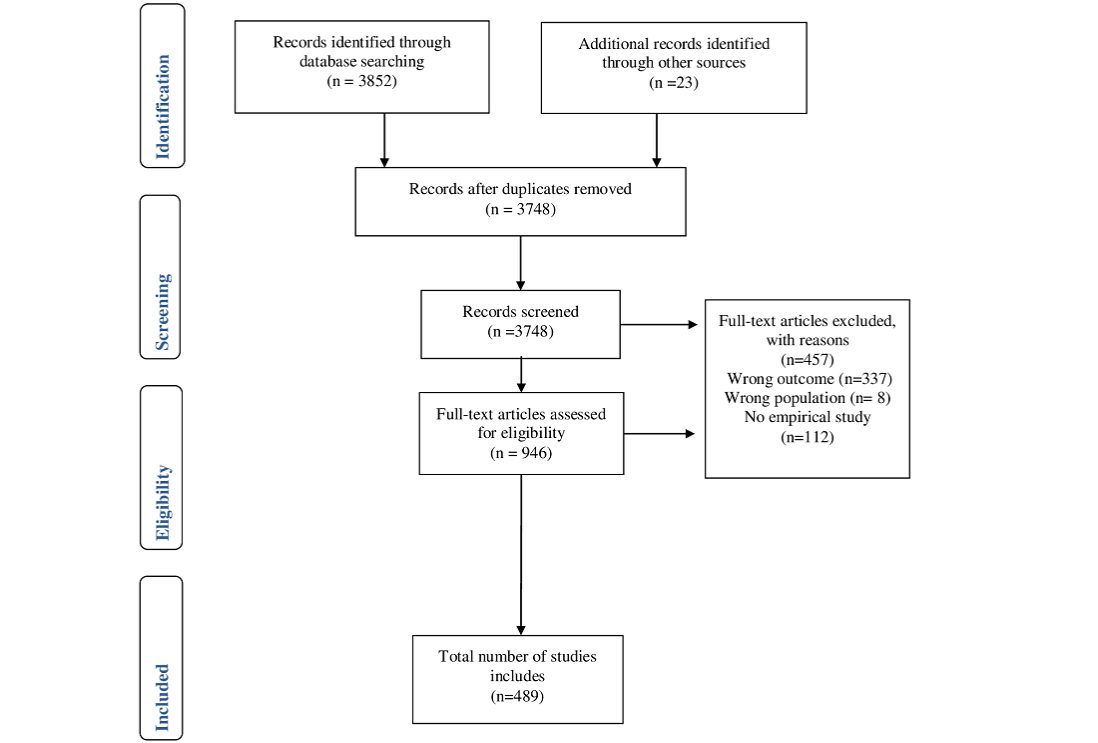
PRISMA (Preferred Reporting Items for Systematic Reviews and Meta-Analyses) flow diagram of search results and study selection.

This scoping review was performed according to the methodological framework as put forward by Khalil et al [18]. As a first step, goal and research question of the scoping review were predefined. Second, in identifying the relevant studies, adjustments to the framework were made; for instance, unlike what has been recommended by Khalil et al [18], no search was performed for gray literature sources for practical and economical research efficiency reasons. PubMed (MEDLINE) and PsycINFO searches were performed by 1 author (UM) on January 20, 2022. Third, the studies were carefully screened in a five-stage procedure by the team of researchers: (1) abstracts were screened in dyads, irrelevant studies were excluded, and duplicates were removed (UM, CH, MP, KPD, JC, EG, and JvS); (2) full texts were screened; (3) further studies were excluded (EG, UM, and CH; Figure 1) and (4) categorized; and finally, (5) the results were collated.

The protocol for this review was registered at OSF Registries on February 19, 2022 [19]. Reporting in this scoping review follows the Preferred Reporting Items for Systematic reviews and Meta-Analyses extension for Scoping Reviews (PRISMA-ScR) checklist (Multimedia Appendix 1) [20]. The checklist contains 20 essential and 2 optional items, following a systematic approach [20]. This checklist was applied to ensure the reporting quality of this review.

### Eligibility

To identify studies for this review, we used the Population, Concept and Context framework by the Joanna Briggs Institute for scoping reviews [21].

The inclusion criteria should meet the Population, Concept and Context framework as follows.

#### Population

This review will include studies that focus on HPs. Only studies conducted with licensed or registered and practicing HPs were included. Studies exclusively conducted in students, trainees, and non–health care professionals or in the general population were excluded.

#### Concept

The concept of interest for this scoping review is health literacy, including the dimensions of *access*, *understand*, *critically appraise*, and *apply* COVID-19–related health information.

We excluded studies focusing on knowledge about professional techniques and methods as well as studies focusing on mental health. Mental health and mental health literacy were outside the scope of this review.

#### Context

The context of this review is the COVID-19 pandemic. All studies from the onset of the COVID-19 (December 2019) pandemic reporting on any dimensional level or facet of COVID-19–related HL with or without explicitly referring to HL and conducted in the context of the COVID-19 pandemic were included. Studies published in peer-reviewed journals and written in English or German language were included. This was a pragmatic decision reflecting the author team’s language proficiency. Moreover, restricting to English language publications appears to have little influence on the introduction of bias in reviews [22,23]. No restrictions were applied regarding study design. Narrative reviews, books or chapters, commentaries, or prefaces were excluded.

### Data Sources and Search Strategy

One of the authors (UM) conducted a search in PubMed (MEDLINE) and PsycINFO on January 20, 2022. All citations were downloaded to Citavi (Swiss Academic Software), and duplicates were removed. The search terms are reported in Multimedia Appendix 2.

### Study Selection

UM, CH, MP, KPD, JC, EG, and JvS independently screened the titles and abstracts for inclusion in dyads. Full texts of the short-listed articles were obtained and independently reviewed in duplicate by 3 authors (EG, UM, and CH), and studies not meeting the inclusion criteria were excluded. Title and abstract screening as well as full-text screening took place in Rayyan (Rayyan Systems Inc) [24].

### Data Collection Process

Three authors (EG, CH, and UM) independently extracted data from the included studies using a pretested data extraction form in Microsoft Excel. Consensus was achieved through discussions and arbitration within the review team.

### Data Items

The included studies were iteratively examined to find items representing the 4 HL dimensions of *access*, *understand*, *critically appraise*, and *apply* COVID-19–related health information. All authors participated in this process, and findings were discussed until consensus was reached.

Ultimately, a total of 10 categories were developed based on these items, which are presented in the results section. In addition, data on study design were extracted.

### Synthesis of Results

Findings were synthesized descriptively and narratively to provide a systematic classification of HL dimensions studied. Furthermore, we provide tables including frequency counts wherever possible. We did not conduct a critical appraisal of the included studies, because it was not within the scope of this review.

## Results

### Selection

The PRISMA (Preferred Reporting Items for Systematic Reviews and Meta-Analyses) flow diagram shown in Figure 1 describes the study selection process. The electronic searches in PubMed (MEDLINE) and PsycINFO and the hand search yielded 3875 references. Following removal of duplicates and the title and abstract screening, 946 full-text articles were assessed for eligibility. A further 457 full-text articles were excluded with reasons, leading to 489 studies finally included in this scoping review. The list of included studies can be found in Multimedia Appendix 3.

### Population

The population included in the studies encompassed HPs such as nurses, pharmacists, community health workers, and medical doctors of most specializations. A clear distinction between the specific HPs studied and the setting in which data collection took place was often not made. Of the 489 included studies, 277 (56.6%) studied HPs, 86 (17.6%) were conducted in dental settings, 45 (9.2%) among nurses, 30 (6.1%) among pharmacy settings, 28 (5.7%) among medical doctors (eg, physicians, surgeons, pediatricians, and general practitioners), the rest (n=22, 4.5%) among a variety of settings such as community health work; emergency medical services; intensive care unit; ear, nose, and throat care; eye care; radiology; and physiotherapy. Only 1 (0.2%) study referred to HPs in a residential home setting.

### Types of Studies

Most of the included studies had used a cross-sectional design. Very few studies reported an intervention aiming to improve a COVID-19–related HL outcome. Of these, only 1 was conducted as a randomized controlled trial; all others used a pre-post design (Table 1). Interventions aimed at improving infection prevention and control knowledge or competencies such as proper PPE application or hygiene measures.

**Table 1:** Study design used in the included studies (N=489).

Study design	Frequency, n (%)
Cross-sectional	415 (85)
Longitudinal	7 (1.4)
Interventional	27 (5.5)
Systematic review	9 (1.8)
Narrative review	1 (0.2)
Qualitative	18 (3.7)
Mixed method	4 (0.8)
Other	8 (1.6)

### Concept and Dimensions of HL

Concept of the scoping review was HL. The authors developed the following categories of HL, based on the four HL dimensions; these categories are as follows: (1) HL (objective versus subjective), (2) sources of COVID-19 information, (3) knowledge (objective versus subjective), (4) ability to understand COVID-19–related information, (5) critically evaluate COVID-19–related information, (6) perceived skills or confidence and perceived preparedness in applying COVID-19–related information, (7) development of educational resource to improve any HL dimension, (8) reported receipt of infection control–related health education training, (9) COVID-19–related HL instrument development, and (10) interventions to improve any of the four HL dimensions.

Almost no included study explicitly referred to or introduced the concept of HL (n=482, 99%). However, all examined studies at least implicitly mentioned one dimension of COVID-19–related HL (Table 2). Of the 489 included studies, the HL dimension of *access* information was investigated by 191 (39.1%) studies; the HL dimension of *understand* information was represented by 434 (88.8%) studies included by a measure of COVID-19–related knowledge; the HL dimension of *critically appraise* information was only examined in 1 (0.2%) study. Moreover, 59 (12.07%) studies measured the HL dimension ability to *apply* COVID-19–related information. The HL dimension *apply* was reviewed using the two categories perceived skills or confidence (n=28, 5.7%) and perceived preparedness (n=31, 6.3%).

COVID-19 knowledge was most frequently examined as objectively measured knowledge. Fewer studies assessed knowledge subjectively, and only 31 (6.3%) studies measured knowledge complementarily by objective and subjective items (Table 3).

Of the 489 studies, 14 (2.9%) reported on the development of an educational training resource to increase (inferred) HL facets. In 148 (30.3%) studies, a measure of reported receipt of infection prevention and control-related training was assessed. Only 7 (1.4%) studies explicitly stated to have addressed HL (Table 4). As the studies presented below show, HL has many possible fields of application.

**Table 2:** Health literacy (HL) dimensions implicitly examined in the included studies (N=489^a^).

HL dimensions		Frequency, n (%)
**Access**		
	Sources of information	191 (39.1)
**Understand**		
	Knowledge (any)	434 (88.7)
	Other than knowledge	13 (2.7)
**Critically appraise**		
	Information	1 (<1)
**Apply**		
	Perceived skills or confidence	28 (5.7)
	Perceived prepared-ness	31 (6.3)

^a^Multiple entries possible; hence, numbers do not add up to 489 or 100%.

**Table 3:** Type of knowledge assessment in the included studies (n=434^a^).

Knowledge assessment	Value, n (%)
Subjective (perceived) knowledge	81 (18.7)
Objective knowledge	280 (64.5)
Subjective (perceived) and objective knowledge	31 (7.1)
Knowledge unclear	42 (9.7)

^a^No multiple entries.

**Table 4:** Studies explicitly referring to health literacy (HL).

Study	Study design or objective	Type of HL	Instrument used	Subjectively or objectively assessed	Validated instrument
Alam et al [25], 2021	Cross-sectional survey	Vaccine literacy	Author developed	Unclear	No
Do et al [26], 2020	Cross-sectional survey	General HL; digital HL	HLS-SF12, eHEALS	Subjective	Yes; partly
Fatteh et al [27], 2022	Cross-sectional survey	COVID-19 HL	Author developed	Subjective and objective	No
Hara et al [28], 2021	Cross-sectional survey	Vaccine literacy	Author developed	Subjective	Partly
Heiniger et al [29], 2021^a^	Instrument development	Hygiene competence	HygiKo; author developed	Objective	Yes
Hiltrop et al [30], 2021	Instrument development	COVID-19 HL	HL-COV-HP; author developed	Subjective	Partly^b^
Nahidi et al [31], 2021	Cross-sectional survey	COVID-19 HL^c^	Author developed	Subjective	Content validated

^a^Referring to objectively assessed competence.

^b^Exploratory and confirmatory analyses conducted in same sample.

^c^HL referred to in Discussion.

### Measurement of COVID-19–Related Health Literacy

A total of 2 (0.4%) studies attempted to develop an instrument, measuring COVID-19–related HL in HPs. Heininger et al [29] developed an objective test, the situational judgement test, HygiKo, to assess hygiene competence. It comprises 20 picture vignettes. Each vignette shows at least one HP and a patient in clinical situations in which hygiene is a pertinent subject. Item-response analyses demonstrated that HygiKo is appropriate for assessing hygiene competence and that it allows distinguishing between persons demonstrating different levels of ability.

Another study [30] developed HLS-COV-HP to measure subjective COVID-19–related HL in HPs. It was adapted from the HLS-EU-Q16 and contains in its present form 12 items to assess the perceived motivation and ability of HPs to find, understand, evaluate, and use COVID-19 information. However, exploratory and confirmatory analyses were performed using the same sample.

A total of 7 (1.4%) studies included an HL measure in an observational survey design. Alam et al [25] examined the motivation to receive a COVID-19 vaccination using a cross-sectional survey design. They also assessed vaccine literacy (VL) by 6 questions from a self-report questionnaire. VL levels were found to differ as a function of gender, age, occupation, or type of organization. The relationship with vaccination motivation was not examined. Do et al [26] evaluated the psychometric properties of an instrument measuring digital HL (eHEALS) and examined associations of subjective general and digital HL with adherence to infection prevention and control procedures among other constructs by conducting a cross-sectional survey in HPs. They found a positive relationship between both general and digital HL, on the one hand, and adherence to infection prevention and control procedures, on the other.

Fatteh et al [27] administered a self-developed questionnaire measuring subjective and objective aspects of COVID-19–related HL to the workforce of a large medical center. They found a positive relationship between medical education level and COVID-19–related HL.

Hara et al [28] conducted a cross-sectional survey in HP and the general population assessing VL and vaccine hesitancy. HPs were found to have higher levels of VL compared with the general population, but the levels of vaccine hesitancy were similar between the groups.

Nahidi et al [31] conducted a cross-sectional survey in critical care nurses. They assessed the ease or difficulty of knowledge acquisition across 11 key information areas of COVID-19, such as the use of PPE, infection prevention and control, or signs and symptoms. Most participants reported a “good” to “very good” level of knowledge about COVID-19 and obtained up-to-date COVID-19 information from a variety of credible sources.

## Discussion

### Overview

This scoping review provides a summary of research on COVID-19–related HL conducted during the COVID-19 pandemic in HPs. HL is considered a key competence to protect oneself, one's patients, but also one's relatives from potential COVID-19 infection; it also entails competencies regarding vaccination-related aspects, detecting infections at an early stage (eg, through regular testing) and seeking medical assistance in case of a positive test or symptoms. Definitions of health literacy by Nutbeam [11] and Sorensen [12] were used to guide the conduct of this scoping review. For a scoping review focusing on health literacy in the context of the pandemic, it seems appropriate to present in more detail those studies that address HL explicitly as a concept. Thus, in the context of this review, we also present a fundus of what an exemplary engagement with HL might explicitly look like. Our results suggest that HL in HPs during a pandemic has rarely been studied in light of a theoretically founded framework of HL. However, a large body of studies measured variables subsumed to be HL dimensions without explicitly referring to HL as a theoretical construct.

### Principal Results

A comprehensive literature search identified 489 studies having examined COVID-19–related HL in HPs. The vast majority, while examining at least one HL dimension, had not intended to study HL as a distinct construct. Of the included studies, only 7 (1.4%) studies explicitly addressed HL. More specifically, 3 (0.6%) studies directly addressed COVID-19–related HL [27,30,31], 2 (0.4%) studies examined general HL in the context of the COVID-19 pandemic [26,32], and another 2 (0.4%) examined vaccination-related HL [25,28]. Digital HL was examined in 1 (0.2%) [26] and hygiene competence in another (n=1, 0.2%) study [29].

Although the overall body of identified studies was heterogeneous, most reviewed studies used a cross-sectional observational survey design to assess among other constructs subjective or objective knowledge related to COVID-19. Because respondents often overestimate their levels of knowledge, competence, or abilities when assessed by subjective self-report [29], it is noteworthy that most of these studies assessed objective knowledge. Some authors [33,34] recommend a complementary assessment of subjective and objective HL because subjective HL should be considered as a separate concept from objective HL [35]. Few studies assessed the HL dimension *understand* by other means beside knowledge. Our analysis found that the HL dimension *access* or *find* information was only represented by the measurement of sources of information. We do not think this can be considered a sufficient approach to measuring this HL dimension. Sources of information was the second most frequently reported dimension in the included studies.

In contrast to the many studies reporting on the HL dimensions *access* and *understand* information, only 1 study reported on the critical appraisal of COVID-19–related information in HPs. Though working in a health care environment, HPs may also be exposed to conflicting information and misinformation. Critical HL is crucial in individuals’ ability to distinguish fact from fake [36].

Approximately 20% of the included studies examined a measure of the HL dimension *apply* COVID-19–related information, by for instance, perceived skills or confidence and perceived preparedness regarding use of infection prevention and control-related measures. It is stressed that the HL dimension *apply* does not pertain to the intention or motivation to enact protective behavior nor the actual behavior itself. Within the HL framework, *apply* (like all other dimensions) is a reflection of the mere abilities or competences rather than the realizations or manifestations of these abilities.

While we came across interventions aiming to improve infection prevention and control-related knowledge and abilities, we found no studies trying to improve vaccination-related knowledge or competencies.

### Limitations

In this scoping review, our systematic search was limited to 2 major databases, and no gray-literature search was conducted. Although it is generally recommended to include gray literature in a scoping review, we decided against including all possible sources for practical and economical research efficiency reasons: The COVID-19 pandemic led to an unprecedented high-speed publication of a large body of scientific literature, both peer-reviewed as well as gray, and to handle this large volume of publications would have required more resources.

Owing to the large number of included studies, we refrained from reviewing the included literature more thoroughly, for instance, regarding quality assessment. This reflects the nature of a scoping review, which is intended to provide a summary of the state of research without addressing the quality of individual studies, according to the standard guidelines for observational or intervention studies.

A more detailed categorization and charting of the HL dimensions may have been beneficial. For instance, we would have liked to provide a more elaborate analysis on the type of COVID-19–related knowledge as knowledge could refer to transmission, course, symptoms, or the prevention of COVID-19.

### Future Research

The observed paucity of research in HPs applying empirically developed HL formulations to pandemic contexts calls for future research. From the current review, many questions remain unanswered. While all areas of HPs’ working environments appeared to have provided studies for our review, it is surprising that only 1 study was conducted among staff in nursing or residential homes. In many countries, these facilities were the ones most hard hit by COVID-19 and should thus be considered more strongly in future research [37].

Our scoping review also revealed that there is a need to use more comprehensive approaches to the measurement of HL dimensions. Altogether, most studies provided very little evidence about the psychometric properties of the used instruments (results not shown). We identified 2 instruments for COVID-19–related HL assessment in HPs, but further validation and refinement appears necessary. There is also a need for instruments objectively measuring a broader range of COVID-19–related HL dimensions in HPs.

Investigations aiming to assess change in HL over time, for instance by repeated surveys attempting to monitor HL levels over the course of the pandemic in HPs, would also be desirable. As there is a need to conduct more robust experimental studies to examine the effectiveness of HL interventions among HPs, such instruments could be used to examine long-term effects of these interventions.

It would probably be profitable for future research to provide more comprehensive reviews, including gray literature and larger bodies of literature by searching more than 2 databases.

### Conclusions

Based on the existing literature on HL in general and related to other health issues, we assume that high levels of COVID-19–related HL among HPs are necessary to ensure not only safe practice with necessary protection of HPs, their patients, and relatives but also successful care delivery. Subsequently, health outcomes may be improved in the long term.

To advance our understanding of how high COVID-19–related HL manifests itself in HPs, how it relates to health outcomes, and how it can be improved, more research is necessary.

## Appendix

Multimedia Appendix 1 PRISMA-ScR (Preferred Reporting Items for Systematic reviews and Meta-Analyses extension for Scoping Reviews) checklist.

Multimedia Appendix 2 Search strategy.

Multimedia Appendix 3 List of included and excluded studies.

## Abbreviations

HL: health literacy
HP: health professional
PPE: personal protective equipment
PRISMA: Preferred Reporting Items for Systematic Reviews and Meta-Analyses
PRISMA-ScR: Preferred Reporting Items for Systematic reviews and Meta-Analyses extension for Scoping Reviews
VL: vaccine literacy
