# Supplementary material for: Health Literacy in Health Professionals Two Years into the COVID-19 Pandemic: Results From a Scoping Review
Source: JMIR Med Educ. 2022 Oct 17;8(4):e39023. doi: 10.2196/39023 (PMC9578515; doi:10.2196/39023)
Supplement: Multimedia Appendix 2 [file mededu_v8i4e39023_app2.docx]

**Multimedia Appendix 1** Search strategy

# Appendix 1. Search strategy for PubMed

| **Population** | ("health personnel"[MeSH Terms] OR ("health"[All Fields] AND "personnel"[All Fields]) OR "health personnel"[All Fields] OR ("personnel"[All Fields] AND "health"[All Fields]) OR "personnel health"[All Fields] OR Pharmac*[Title/Abstract] OR Ophthalmol*[Title/Abstract] OR dent*[Title/Abstract] OR nurse*[Title/Abstract] OR surgeon*[Title/Abstract] OR (medical AND professionals [Title/Abstract]) OR "healthcare workers" [Title/Abstract] OR "healthcare providers" [Title/Abstract] OR "health care providers" [Title/Abstract] OR "health professionals" [Title/Abstract] OR "health care professionals" [Title/Abstract]) |
| --- | --- |
| Phenomenon of **interest** | (health literacy[MeSH Terms] OR (health[Title/Abstract] AND competen*[Title/Abstract]) OR literacy[Title/Abstract] OR knowledge[Title/Abstract] OR skills[Title/Abstract] OR (health[Title/Abstract] AND (capabilit*[Title/Abstract] OR abilit*[Title/Abstract]))) |
| **Context** | ((2019-nCoV OR 2019nCoV OR COVID-19 OR SARS-CoV-2 OR ((wuhan AND coronavirus) AND 2019/12[PDAT]:2030[PDAT])) OR ("Severe Acute Respiratory Syndrome"[MeSH Terms])) |
| Not | (animals [mh] NOT humans [mh]) |
